# Intrinsic Frontolimbic Connectivity and Mood Symptoms in Young Adult Cannabis Users

**DOI:** 10.3389/fpubh.2019.00311

**Published:** 2019-11-01

**Authors:** Skyler Shollenbarger, Alicia M. Thomas, Natasha E. Wade, Staci A. Gruber, Susan F. Tapert, Francesca M. Filbey, Krista M. Lisdahl

**Affiliations:** ^1^Psychology Department, University of Wisconsin-Milwaukee, Milwaukee, WI, United States; ^2^Department of Psychiatry, University of California, San Diego, La Jolla, CA, United States; ^3^Imaging Center, McLean Hospital, Belmont, MA, United States; ^4^Department of Psychiatry, Harvard Medical School, Boston, MA, United States; ^5^Bert Moore Chair in BrainHealth, School of Behavioral and Brain Sciences, The University of Texas at Dallas, Dallas, TX, United States

**Keywords:** cannabis, resting state fMRI, young adults, adolescents, affective symptoms, depressive symptoms, connectivity analysis

## Abstract

**Objective:** The endocannbinoid system and cannabis exposure has been implicated in emotional processing. The current study examined whether regular cannabis users demonstrated abnormal intrinsic (a.k.a. resting state) frontolimbic connectivity compared to non-users. A secondary aim examined the relationship between cannabis group connectivity differences and self-reported mood and affect symptoms.

**Method:** Participants included 79 cannabis-using and 80 non-using control emerging adults (ages of 18–30), balanced for gender, reading ability, and age. Standard multiple regressions were used to predict if cannabis group status was associated with frontolimbic connectivity after controlling for site, past month alcohol and nicotine use, and days of abstinence from cannabis.

**Results:** After controlling for research site, past month alcohol and nicotine use, and days of abstinence from cannabis, cannabis users demonstrated significantly greater connectivity between left rACC and the following: right rACC (*p* = 0.001; corrected *p* = 0.05; *f*^2^ = 0.55), left amygdala (*p* = 0.03; corrected *p* = 0.47; *f* ^2^ = 0.17), and left insula (*p* = 0.03; corrected *p* = 0.47; *f* ^2^ = 0.16). Among cannabis users, greater bilateral rACC connectivity was significantly associated with greater subthreshold depressive symptoms (*p* = 0.02).

**Conclusions:** Cannabis using young adults demonstrated greater connectivity within frontolimbic regions compared to controls. In cannabis users, greater bilateral rACC intrinsic connectivity was associated with greater levels of subthreshold depression symptoms. Current findings suggest that regular cannabis use during adolescence is associated with abnormal frontolimbic connectivity, especially in cognitive control and emotion regulation regions.

## Introduction

Cannabis remains one of the most popular used substances worldwide (1). Approximately, 35% of high school seniors and young adults ages 19–28 reported using cannabis in the past year (2). Cannabis use during youth has been a recent focus in public health research, as it may influence one's risk for reporting symptoms of anxiety and depression (3–14). A potential mechanism underlying cannabis' influence on mood and affective symptoms may involve frontolimbic functioning [see (15, 16)]. Understanding differences in frontolimbic connectivity among young adults with frequent cannabis use may provide insight into the etiology of associated mood or affective risk.

Cannabinoids in cannabis, such as Δ9-tetrahydrocanabidiol (or THC) and cannabidiol (CBD), are chemicals that mimic endogenous neurotransmitters anandamide and 2AG by binding to endocannabinoid (eCB) receptors CB_1_ and CB_2_ (17–20). THC is the main psychoactive component of cannabis and is responsible for the subjective “high” individuals experience [see (20–22)]. CB_1_ activity modulates the release of the neurotransmitters GABA and glutatmate (GLUT) [see (23)]. The eCB system modulates several functions related to physical (e.g., sleep, pain, and inflammation) and mental health, including regulation of emotional and stress responses [see (24–29)].

More specifically, the eCB system plays a role in mood and affect (28, 30–35), integrating reward feedback (36), and threat related signals (37–39). Brain regions primarily involved in the affective processing system include several interacting cortical and subcortical regions (e.g., amygdala, anterior cingulate gyrus or ACC, medial and inferior orbito-frontal, ventromedial or vmPFC, dorsomedial prefrontal cortex, ventral striatum, and insula) (40–44). This system is highly innervated with CB_1_ receptors (45–49) and animal models demonstrate developmental changes in CB_1_ expression within the mPFC, ACC and insula (50), suggesting the system demonstrates plasticity during adolescence. Therefore, repeated THC exposure during development may impact naturally occurring changes in eCB functioning within mesocorticolimbic regions (16). Indeed, daily cannabis users have shown decreased CB_1_ receptor density within frontolimbic regions (prefrontal cortex (PFC), ACC, and insula) compared to non-users which recovered after a month of abstinence (51). Further, acute THC administration has resulted in abnormal performance on behavioral measures of emotional processing (52–54), amygdala reactivity (38), and altered functional connectivity and signaling in PFC regions (15, 16, 53, 55–58). However, additional research is needed to confirm the influence of repeated THC exposure on affective outcomes in adolescents and young adults.

Due to the neuromodulatory role of the eCB system, examining brain functional connectivity is an important outcome to study in regular cannabis users. These relationships can be examined during tasks and also at rest, when individuals are not actively engaging in any specific cognitive tasks, called resting state, or intrinsic functional connectivity (ifcMRI) (59). Connectivity patterns in frontolimbic regions continue to develop into late adolescence and emerging adulthood; prefrontal maturation purports enhanced emotion regulation and behavior inhibition capabilities [see (60–68)], giving rise to a functional coupling between frontal and limbic regions (i.e., the frontolimbic network) (69). Collectively, the developmental changes in frontolimbic connectivity are thought to enhance socioemotional regulation [see (70–72)], specifically via functioning within the amygdala, medial PFC, vmPFC, ACC, insula, and inferior frontal gyrus (43, 73). A particular region within the PFC, the ACC, also undergoes significant age-related changes in intrinsic functional connectivity, particularly in rostral ACC (rACC) subregions involved in social cognition and emotion regulation (74). Therefore, this system may be particularly vulnerable to repeated THC exposure during development.

Thus far, studies have found slower response times in users when identifying emotional faces and more liberal criterion for selecting sadness (75), poorer facial recognition and emotion matching (76), and emotion identification and discrimination impairments (77) compared to non-users; though accuracy in emotion identification may not display a dose-dependent relationship (78). fMRI studies have found aberrant amygdala and ACC activity in young cannabis users during affective processing tasks, including blunted ACC and amygdala activation during sub-conscious facial viewing (79), blunted amygdala response among youth with comorbid cannabis dependence and depression (80), and greater amygdala reactivity to angry faces in young adolescents (81).

However, to date very few studies have examined intrinsic functional connectivity (ifcMRI) in adolescents and emerging adults (82–86). Studies to date in adolescent and young adult cannabis users (primarily male) have demonstrated increased intrinsic connectivity in frontal (superior, inferior frontal gyrus)-temporal gyrus-cerebellar regions (83), frontal-parietal-cerebellar network (84), increased middle-frontal and cingulate gyrus connectivity (85), and increased frontal gyrus activity along with reduced middle temporal activity (82). Increased connectivity patterns were linked with increased symptoms of cannabis dependence (83) and recent cannabis use frequency (84). In young adult males, cannabis use was linked with increased connectivity in insula and decreased connectivity in the anterior cingulate and midbrain, even after a month of abstinence (86). Thus, overall, young cannabis users appear to demonstrate increased intrinsic connectivity patterns, especially in frontal-limbic regions. Still, these studies were primarily in men (83, 84, 86), thus findings may not generalize to female users (87–90). Further, two studies did not control for comorbid alcohol use (83, 86) and despite the aforementioned link between cannabis use and affective processing, no studies to date have specifically examined affective processing networks in cannabis users. Therefore, additional research is needed to examine intrinsic connectivity in affective processing networks in larger samples that include both males and females, controlling for comorbid alcohol use.

The purpose of the current study was to explore whether regular cannabis use in adolescents and young adults was associated with aberrant ifcMRI frontolimbic connectivity at rest. We employed a priori region of interest analysis focusing on regions with reported cortical differences between young cannabis users and controls, including: vmPFC (91, 92), ACC (81, 93, 94), insula (95), and amygdala (88, 96, 97). This project utilized ifcMRI data from three collection sites from the Imaging Data in Emerging Adults with Addiction (IDEAA) Consortium (University of Wisconsin-Milwaukee or UWM; McLean Hospital/Harvard University or McLean; University of Texas—Dallas or UTD). The strength of utilizing multi-site data sets include excellent reliability and validity when combining multi-site ifcMRI data (98–107), increased generalizability of more heterogenous groups (i.e., improving sex, ethnicity, and geographic diversity), and larger sample sizes. It was hypothesized that cannabis users would demonstrate increased intrinsic connectivity patterns in regions subserving emotional expression [amygdala, insula, and caudal (cACC) and rostral ACC (rACC)]. Lastly, in order to interpret the findings, a secondary aim examined if group differences in connectivity were associated with cannabis users' self-reported anxiety and depressive symptoms.

## Materials and Methods

### Participants

Participants included 79 cannabis users (42 men and 37 women) and 80 (45 men and 35 women) controls aged 18–30 year old young adults devoid of major medical, psychiatric or neurologic comorbidities. This age restriction is to reduce potential differences in developmental stage since adolescents and emerging adults may have greater PFC and limbic development compared to adult participants (60–64, 68, 108). Study participants were selected from the IDEAA consortium subject pool (PIs: Krista Lisdahl, Ph. D., UWM; Staci Gruber, Ph. D., McLean Hospital/Havard, Susan Tapert, Ph.D., University of California-San Diego, and Francesca Filbey Ph.D., UTD; data from Dr. Tapert's lab did not include resting-state fMRI collection and therefore was not used in the current study).

*Inclusion criteria* included: right-handedness; had usable intrinsic ifcMRI data; fluency in English; and fit one of two groups: cannabis users (at least weekly cannabis use within the past 3 months, duration of use >1 year) and controls (never had a history of regular (>monthly) use; no recent past month use; no history of cannabis use disorder). *Exclusion criteria* included history of neurological illness or loss of consciousness >2 min; MRI contraindications (pregnancy, claustrophobia, weight over 250 lbs., ferromagnetic implants of any kind, pacemakers, or other devices in body); current use of psychoactive medication; current DSM-IV-TR (109) independent Axis I disorders (aside from substance use disorders); regular other illicit drug use (>20 times); and inability to remain abstinent from all drugs and alcohol for at least 12 h (ranged from 12 h to 21 days monitored abstinence across sites).

### Procedures

The Institutional Review Board for each site approved all aspects of data collection. Participants underwent site-specific IRB-approved consenting procedures, and completed screening sessions to ensure inclusion/exclusion criteria. Following study inclusion, the participants completed psychological questionnaires, underwent substance toxicology screening, and received an MRI at the individual collection sites. The ifcMRI data was collected before any fMRI task for each site.

### Inventories and Questionnaires

#### Substance Use

Drug use prior to study participation was recorded by interview using temporal memory cues from a modified version of the Time-Line Follow-Back at each study site (110). Drug categories included quantity-standardized collection of: nicotine cigarettes (total number), alcohol (total standardized drinks), cannabis (total number of grams of dried flower^1^), and other illicit drugs (days used). Time-period covered for substance use assessment was 1 month [original data collected from each site ranged from 2 weeks (McLean), past 30 days (UTD), to past year (UWM); thus, total *past month* substance use was averaged for each participant collected from McLean, though all McLean users reported consistent daily patterns of use during this time].

#### Depressive Symptoms

The Beck Depression Inventory—second edition (BDI-II) (collected from all sites) measured self-reported symptoms of past 2-week depressive symptoms with a possible range of 0–63 total scores (111, 112). Low scores on the BDI-II are interpreted as ≤ 16 and elevated ≥17.

#### Estimated Verbal IQ

The Wechsler Abbreviated Scale of Intelligence (WASI)—Vocabulary subtest (113) (collected from McLean and UTD) and the Wide Range Achievement Test−4th edition (WRAT-IV). Reading subtest (114) (collected from UWM) measured verbal intelligence (115) and quality of education [see (116)]. Standardized (age-corrected) T-scores for each participant were used in the analyses.

### MRI Acquisition and Preprocessing

#### MRI Acquisition Parameters

Image processing followed standardized recommendations for fMRI processing (117, 118). ifcMRI scans were combined from three research sites; de-identified raw DICOM files were uploaded to the McLean Hospital server. *UWM*: Structural MRI (sMRI) scans were collected using a 3T GE MR750 scanner and SPGR sequence with the following parameters: TR/TE/TI = 8.2/3.4/450 ms, flip angle = 12°, FOV = 240, matrix size: 256 × 256 mm, slice thickness = 1 mm (along left-right direction), voxel size = 1 × 1 × 1 mm, 150 slices, total scan time = 8 min. ifcMRI scans were collected using a gradient echo, echoplanar sequence with ramp sampling correction using the intercomissural line (AC-PC) as a reference (TR: 2,000 ms, TE: 25 ms, FOV: 240, flip angle = 77°, matrix size: 64 × 64, 40 slices, reps: 240, thickness 3.7 mm). *McLean*: sMRI scans were collected using a 3T Siemens Magnetom TrioTim sngo MR B17 and MPRAGE sequence with the following parameters: TR/TE/TI = 2,000/2.15/1,100 ms, flip angle = 12°, FOV = 256 × 256 mm, slice thickness = 1.33 mm (along left-right direction), voxel size = 1.5 × 1.0 × 1.3 mm, total scan time = 9 min. ifcMRI scans were collected using a gradient echo, echo-planar sequence (TR: 2,500 ms, TE: 30 ms, flip angle: 82° degrees, matrix size: mm, 41 slices, voxel size: 3.5 × 3.5 × 2.5 mm^3^). *UT Dallas*: sMRI images were collected using a 3T Philips whole body scanner equipped with Quasar gradient subsystem (40 mT/m amplitude, a slew rate of 220 mT/m/ms). A 32-channel receive head phased array coil combined with body coil transmission to achieve greater sensitivity in cortical areas. sMRI scans utylized an MPRAGE sequence with the following parameters: TR/TE/TI = 2,100/3.70/1,100 ms, flip angle = 12°, FOV = 256 × 256 mm, slab thickness = 160 mm (along left-right direction), voxel size = 1 × 1 × 1 mm, total scan time = 3 min 57 s. fMRI scans were collected using a gradient echo, echo-planar sequence with the intercomissural line (AC-PC) as a reference (TR: 2.0 s, TE: 29 ms, flip angle: 75 degrees, matrix size: 64 × 64, 39 slices, voxel size: 3.44 × 3.44 × 3.5 mm^3^).

#### Preprocessing Details

All images were preprocessed utilizing an identical pipeline, computing system, and software versions (no updates were conducted during data analysis) at UWM. *Anatomical preprocessing* utilized the CPAC analysis software for large multisite datasets (see: https://fcp-indi.github.io/), which utilized pre-existing imaging software, including AFNI (119), FSL (120), and ANTS (http://stnava.github.io/ANTs/). Data were deobliqued to align with X, Y, and Z coordinates; resampled to FSL friendly RPI anatomical convention; skull stripped; anatomical segmentation; and binarized threshold masks were created utilizing FSL's FAST; functional images were linearly registered to anatomical native space using FSL's FLIRT; anatomical images underwent non-linear transformation to MNI152 (voxel size = 2 mm^3^) standard brain template using ANTS. *fMRI* was also preprocessed using the CPAC software using the following steps: removal of the initial 5 time points to allow T1 stabilization; deoblique; resampling to RPI space; skull stripping; data was “scrubbed” using Framewise Displacement (121) with a maximum TR displacement set to 4 mm; image intensity normalization; linear and quadratic detrending to remove residual drift due to scanner heating and/or slower head movement; nuisance regression (white matter and cerebrospinal fluid) using 6 displacement and motion correction parameters using CompCor (applied prior to smoothing); spatial smoothing (Gaussian Kernal = 4 mm FWHM; Sigma = 2.54); and temporal filtering (Band Pass filter = 0.1–0.01 Hz). *Frontolimbic ROI's*. Cortical and subcortical ROI's were created using FreeSurfer's (122) cortical parcellation atlas [DKT40 atlas; (123)] and subcortical segmentation (124). ROI's included the bilateral rostral anterior cingulate (rACC), caudal anterior cingulate (cACC), ventral medial PFC (vmPFC), insula, and amygdala.

### Data Analysis

#### fMRI Data Analysis; Primary Aim 1

For each subject, the average time series was extracted for all aforementioned ROI's using the CPAC software. Next, the correlation coefficients for the time series were created using MATLAB (Version 8.0.0.783 64-bit maci64, 2012). Lastly, a series of standard multiple regressions were run to predict correlation coefficients between each set of ROIs; the primary predictor variable (cannabis group status), and covariates (past month nicotine use, past month alcohol use, MRI collection site, and duration of abstinence from cannabis prior to scan) were entered utilizing standard least squares multiple regression in SPSS (version 24). Specifically, the first block included all covariates (past month nicotine use, past month alcohol use, behavioral/MRI collection site, and duration of abstinence) and the second block included cannabis group status. False Discovery Rate correction [FDR; (125)] was implemented to correct for multiple comparisons. All correlation coefficients between ROIs were visually inspected for normality in distribution. Skewed distributions were transformed using a log_10_ transformation and used in the regression in place of the skewed correlation coefficients. There was no evidence of multicollinearity or homoscedasticity following inspection of the standardized residual for the variables of interest. Interpretations of statistical significance were made if *p* < 0.05. For ease of interpretation, regions with connectivity differences after correction for multiple comparisons were also displayed on an average template brain provided by BrainNet Viewer software [(126); see **Figure 2** below].

#### Brain-Behavior Relationships: Secondary Aim

Pearson r correlations were run between connectivity coefficients and total depressive symptoms among cannabis users (in regions predicted by cannabis use).

## Results

### Demographic Variables

ANOVAs and χ^2^'s tests examined whether cannabis users and controls differed in demographic variables (see Table 1). Cannabis users and controls did not differ in age [F(1, 157) = 1.1, *p* = 0.3], ethnicity group [64.6% Caucasian for cannabis users and 52.5% for controls, χ^2^ (1)2.4, *p* = 0.12], gender [46.8% female for cannabis users and 43.8% for controls, χ^2^ (1)0.15, *p* = 0.7], and premorbid intelligence [F(1, 156) = 0.46, *p* = 0.5].

**Table 1:** Demographics by group status.

	**Cannabis users (*n* = 79)**	**Controls (*n* = 80)**
Age	23.4 (3.4) [18–30]	22.9 (2.6) [18–29]
Premorbid intelligence Reading standardized score	53.1 (9.7) [31–74]	54.1 (8.9) [30–72]
Gender (% female)	46.8%	43.8%
% Caucasian	64.6%	52.5%
Beck depression inventory (BDI-II) total-2	7.1 (9.3)^*^ [0–53]	3.9 (4.4)^*^ [0–19]
Past month cannabis use Total grams	57.9 (54.3)^**^ [0–217.5]	0 (0)^**^ [0]
Past month total cigarettes Total number	6.9 (22.1)^**^ [0–121]	0.24 (1.4)^**^ [0–12]
Past month alcohol use Total standard drinks	22.3 (28.4)^**^ [0–137]	7.1 (10.9)^**^ [0–62.5]

* p < 0.05 and

** *p < 0.01*.

### Substance Use

As expected, cannabis users differed from controls in past month total grams [F(1, 157) = 91.1, *p* < 0.01], past month total days of cannabis use [F(1, 85) = 9,208.4, *p* < 0.01], past month total standard alcohol drinks [F(1, 157) = 20, *p* < 0.01], and past month total cigarettes [F(1, 157) = 7.3, *p* = 0.01]. The cannabis users were abstinent from cannabis for 12–24 h (27.8%); 2–3 days (39.2%); 4–7 days (5.1%); or 8 days or greater (27.8%).

### Depressive Symptoms

Cannabis users reported significantly greater total BDI-II [F(1, 124) = 5.7, *p* = 0.02] scores compared to controls, although both groups' total BDI-II scores remained in the subclinical range.

### Primary Aim: ROI Intrinsic Connectivity

**Figure 1:**
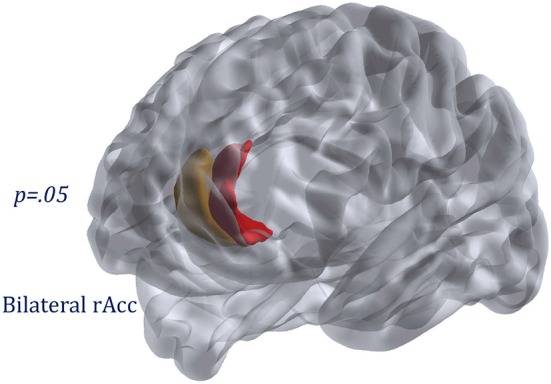
Region displaying group differences: bilateral rostral anterior cingulate.

After controlling for MRI collection site, past month alcohol and cigarette use (in standard units), and days abstinent from cannabis, cannabis users demonstrated significantly increased connectivity between left rACC and the following: right rACC [*t*(80) = 3.3, *beta* = 0.59, *p* = 0.001; FDR corrected *p* = 0.05; Cohen's *f*^2^ = 0.55], left amygdala [*t*(80) = 2.2, *beta* = 0.45, *p* = 0.03; FDR corrected *p* = 0.47; Cohen's *f* ^2^ = 0.17], left insula [*t*(80) = 2.2, *beta* = 0.45, *p* = 0.03; FDR corrected *p* = 0.47; Cohen's *f* ^2^ = 0.16]. There were no group differences where cannabis users demonstrated significant decreases in connectivity compared to controls (see Figure 1 for an image of the bilateral rACC).

#### Brain-Behavior Relationships

**Figure 2:**
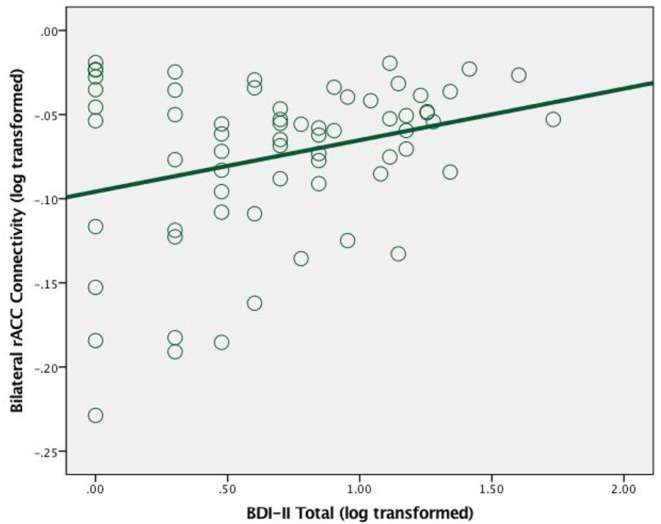
Scatterplot between total depression symptoms and bilateral rAcc connectivity (log transformed) in cannabis users.

Among cannabis users, greater bilateral rACC connectivity was significantly associated with greater total depressive symptoms [*r* = 0.29, *n* = 66, *p* = 0.02] (see Figure 2).

## Discussion

The current study examined whether cannabis use was associated with frontolimbic intrinsic connectivity using a cross-sectional design in a sample devoid of independent Axis I anxiety or mood disorders. After controlling for MRI collection site, recent alcohol, and nicotine use, and abstinence from cannabis use, cannabis users demonstrated increased intrinsic connectivity between the left rACC and the following: left insula, left amygdala, and right rACC in comparison to controls, though only group differences between bilateral rACC survived after correcting for multiple comparisons. Further, we found that increased bilateral rACC connectivity was associated with greater subclinical depressive symptoms in cannabis users.

Current findings parallel previous intrinsic functional studies indicating frequent cannabis use among youth is associated with greater connectivity between frontal and temporal regions (83), and increased ACC connectivity in males (85). Resting state connectivity increases in comparison to controls was also reported within the medial frontal gyrus among a high-risk mostly male adolescent group (82). The present study adds to existing literature by including more females, controlling for other substance use and cannabis abstinence period, and relating the observed connectivity differences to mood-related symptoms. Task-based studies also report altered medial PFC activity associated with cannabis use among emerging adults (79, 127–134), suggesting chronic cannabis use is associated with region-specific changes in brain activity and connectivity among regions implicated in emotion regulation, identification, and modulation.

The current findings of abnormal functional connectivity in the rACC and limbic regions, which is consistent with our previous structural findings. Our team recently reported that greater cannabis use was related to reduced left rACC volume among young cannabis users, and smaller rACC volumes were also significantly associated with lower performance in an emotional discrimination task (94). Further, we also found reduced right ACC cortical thickness in a sample of young cannabis users, including a subset of cannabis users with a history of childhood attention deficit hyperactivity disorder, compared to non-using controls (93). The ACC undergoes significant developmental shifts in functional connectivity during young adulthood (74), has been implicated in ones' ability to detect and monitor self-produced errors (135, 136) whether one is conscious/aware of the error or not (137, 138). The ACC may be less engaged in cannabis users compared to controls during tasks requiring inhibitory control and error monitoring (131). The rostral subdivision of the ACC is functionally connected with the amygdala (139), forming a network for processing affective facets of behavior (140, 141). In concert with the insula, the ACC also serves to incorporate perceptual information with autonomic and emotional information (142). More specifically, the rACC has been posited to have top-down control influence, serving as a gatekeeper, between regions processing negative affective information and those integrating environmental stimuli [see (143, 144)], and demonstrates protracted development during young adulthood (74). The rACC is involved in implicit or automatic emotion regulation that occurs at a subconscious level (42). Indeed, lesions in the rACC are posited to impair ones' sensitivity to adjustments in personal performance during a cognitive control task (145). For example, cannabis users have demonstrated reduced P300 (attention to emotion) during implicit and empathic emotional processing paradigms, particularly for the highest using cannabis users that also demonstrated deficits in explicit processing of negative emotions (146). Thus, abnormalities in rACC structure and function may impact various behavioral aspects, including cognitive control and emotional regulation.

The current study suggests that chronic cannabis use may increase intrinsic connectivity between emotion regulation regions, which was opposite of our original hypothesis. A potential interpretation may include the inefficiency of prefrontal top-down regulation, as hypothesized by Behan et al. (84), suggesting reduced intrinsic amygdala responsiveness. Further, Pujol et al. (86) found reduced ACC and insula connectivity; however, the study did not examine subcomponents of the ACC and used seed-based rather than region of interest approaches. Thus, disruptions in rACC function may lead to challenges in modulating ones' mood, consistent with the current study findings, or adjusting to emotionally salient internal and external information. Indeed, we also found that increased depressive symptoms among cannabis users were associated with greater connectivity between the bilateral rACC. Alterations in rACC structure (147–150) and function [see (151–153)] have been previously linked with depressive and affective symptoms and antidepressant resonse (154). Though the current sample did not meet criteria for an Axis I mood or anxiety disorder, cannabis use may impact regions implicated in symptom manifestation. Although cannabis users reported significantly greater subclinical levels of depression, we are unable to determine whether the endorsed symptoms predated the initiation of cannabis use or whether the endorsed symptoms occurred during the course of regular cannabis use among users. Indeed, cross-sectional (8, 11–13) and longitudinal (5, 6, 13, 99, 155) studies among cannabis-using youth have found increased risk of mood and affective symptoms. Even casual cannabis using young adults report greater depressive symptomatology (156). Thus, structural and functional abnormalities within the rACC observed in cannabis users may result in mood dysregulation. Alternatively, subtle mood dysregulation may be a risk-factor for riskier cannabis use consumption.

Proposed theories accounting for these functional and behavioral differences in cannabis users may have multiple underlying etiologies. Chronic young adult cannabis users demonstrate abnormal CB_1_ receptor density in the ACC (51); thus, frequent cannabis use may influence continued white matter myelination and gray matter pruning within this region, impacting structural integrity (81, 91, 93, 157). Further, altering CB_1_ availability and eCB signaling may impact GABA and GLUT signaling, which is observed in the ACC of adolescents with chronic cannabis use (158, 159), suggesting continued cannabis use may impact healthy ACC functioning. Indeed, rACC glutamate levels have been associated with interactions between task-positive (supragenual ACC) and task-negative (perigenual ACC) subregions (160), suggesting excitatory activity at rest may alter one's ability to engage networks involved in environmental interaction. Thus, altered inhibitory eCB activity may account for changes in intrinsic ACC connectivity among users. It is also possible that abnormalities in rACC and increased symptoms of depression place adolescents and young adults at increased risk for regular cannabis use. Prospective longitudinal studies are needed to address causality.

In terms of youth treatment, there are potential interventions that may target ACC functioning to improve emotional regulation and mood in cannabis users. For example, activation within the ACC was associated with positive treatment outcomes following change talk among a diverse group of cannabis-using youth (161). Mindfulness-based mediation and a combination of mindfulness with aerobic exercise have also been associated with ACC specific changes [see (162)].

Findings from the current study should be considered in light of potential limitations. Although comorbid use of nicotine products was measured, some participants may have smoked cannabis with nicotine mixed in (e.g., blunts); this was not measured in the current study. Given the cross-sectional nature of the current study, potential differences in frontolimbic connectivity and subclinical mood symptoms may exist *prior to* the onset of frequent cannabis use and serve as a risk factor for regular cannabis use during adolescence (163, 164). The relationships between such factors and substance use patterns among youth have previously been investigated [see (165–175)]. Therefore, prospective, longitudinal studies are necessary to determine timing and causality.

In conclusion, the present multisite imaging study found that among otherwise healthy young adults devoid of independent mood or affective disorders, regular cannabis users had greater intrinsic connectivity between left and right rACC. The current study also found that greater intrinsic bilateral rACC connectivity was associated with greater subthreshold depressive symptoms among cannabis users. Results coincide and expand upon prior intrinsic and task-based imaging projects among young adults with chronic cannabis use, suggesting altered connectivity between regions with high cannabinoid receptor density that are imperative for emotional inhibition, recognition, and regulation. As THC content continues to rise (176–178), today's users may be at increased risk for elevated mood or anxiety symptoms (179–181). Considering these findings, it is recommended that youth delay regular use of cannabis until after peak brain maturation is achieved [see (182)]. In light of the current paper, cannabis interventions for youth may target improving anterior cingulate functioning, including aerobic exercise and mindfulness-based approaches [see (162, 183, 184)].

## Ethics Statement

This study was carried out in accordance with the recommendations by the Institutional Review Board at each institution (UWM, McLean, and UTD). All subjects gave written informed consent in accordance with the Declaration of Helsinki.

## Author Contributions

SS developed the aims and hypotheses, assisted with the design, pre-processed the fMRI scans, and conducted the analyses. AT assisted with the MRI preprocessing and data analyses. NW assisted with the IDEAA data management and merging, MRI pre-processing, and edited the manuscript. SG, ST, and FF were an IDEAA site PI, assisted with the design and data analysis, and edited the manuscript. KL was an IDEAA site PI, assisted with development of the aims and hypotheses, and supervised all MRI pre-processing, design, data analyses, and edited all versions of the manuscript.

### Conflict of Interest

The authors declare that the research was conducted in the absence of any commercial or financial relationships that could be construed as a potential conflict of interest.

## Footnotes

^1^Cannabis concentrate usage was very low in the sample (*n* = 3); cannabis concentrates were converted into estimated equivalent grams of flower.

## References

[1] Kuddus M Ginawi IM Al-Hazimi A Cannabis sativa: an ancient wild edible plant of India. Emirates J Food Agric. (2013) 25:736–45. 10.9755/ejfa.v25i10.16400

[2] Miech RA Johnston LD O'Malley PM Bachman JG Schulenberg JE Monitoring the Future National Survey Results on Drug Use 1975-2016: Volume I, Secondary School Students. Ann Arbor, MI: Institute for Social Research, The University of Michigan (2017).

[3] McGee R Williams S Poulton R Moffitt T. A longitudinal study of cannabis use and mental health from adolescence to early adulthood. Addiction. (2000) 95:491–503. 10.1046/j.1360-0443.2000.9544912.x 10829326

[4] Degenhardt L Hall W Lynskey M. The relationship between cannabis use, depression and anxiety among Australian adults: findings from the National Survey of Mental Health and Well-Being. Soc Psychiatry Psychiatr Epidemiol. (2001) 36:219–27. 10.1007/s001270170052 11515699

[5] Brook DW Brook JS Zhang C Cohen P Whiteman M. Drug use and the risk of major depressive disorder, alcohol dependence, and substance use disorders. Arch Gen Psychiatry. (2002) 59:1039–44. 10.1001/archpsyc.59.11.1039 12418937

[6] Fergusson DM Horwood LJ Swain-Campbell N. Cannabis use andpsychosocial adjustment in adolescence and young adulthood. Addiction. (2002) 97:1123–35. 10.1046/j.1360-0443.2002.00103.x 12199828

[7] Patton GC Coffey C Carlin JB Degenhardt L Lynskey M Hall W. Cannabis use and mental health in young people: cohort study. BMJ. (2002) 325:1195–8. 10.1136/bmj.325.7374.1195 12446533 PMC135489

[8] Rey JM Sawyer MG Raphael B Patton GC Lynskey M. Mentalhealth of teenagers who use cannabis: results of an Australian survey. Br J Psychiatry. (2002) 180:216–21. 10.1192/bjp.180.3.216 11872513

[9] Georgiades K Boyle MH. Adolescent tobacco and cannabis use: young adult outcomes from the Ontario Child Health Study. J Child Psychol Psychiatry. (2007) 48:724–31. 10.1111/j.1469-7610.2007.01740.x 17593153

[10] Hayatbakhsh MR Najman JM Jamrozik K Mamun AA Alati R Bor W. Cannabis and anxiety and depression in young adults: a large prospective study. J Am Acad Child Adolesc Psychiatry. (2007) 46:408–17. 10.1097/chi.0b013e31802dc54d 17314727

[11] Wittchen H Fröhlich C Behrendt S Günther A Rehm J Zimmermann P. Cannabis use and cannabis use disorders and their relationship to mental disorders: a 10-year prospective-longitudinal community study in adolescents. Drug Alcohol Dependence. (2007) 88:S60–70. 10.1016/j.drugalcdep.2006.12.013 17257779

[12] Fleming CB Mason WA Mazza JJ Abbott RD Catalano RF. Latent growth modeling of the relationship between depressive symptoms and substance use during adolescence. Psych Addict Behav. (2008) 22:186–97. 10.1037/0893-164X.22.2.186 18540716

[13] de Graaf R Radovanovic M van Laar M Fairman B Degenhardt L Aguilar-Gaxiola S. Early cannabis use and estimated risk of later onset depression spells: epidemiologic evidence from the population-based world health organization world mental health survey initiative. Am J Epidemiol. (2010) 172:149–59. 10.1093/aje/kwq096 20534820 PMC2915487

[14] Degenhardt L Coffey C Romaniuk H Swift W Carlin JB Hall WD. The persistence of the association between adolescent cannabis use and common mental disorders into young adulthood. Addiction. (2013) 108:124–33. 10.1111/j.1360-0443.2012.04015.x 22775447

[15] Egerton A Allison C Brett RR Pratt JA. Cannabinoids and prefrontal cortical function: insights from preclinical studies. Neurosci Biobehav Rev. (2006) 30:680–95. 10.1016/j.neubiorev.2005.12.002 16574226

[16] Ellgren MM Artmann AA Tkalych OO Gupta AA Hansen HS Hansen SH. Dynamic changes of the endogenous cannabinoid and opioid mesocorticolimbic systems during adolescence: THC effects. Eur Neuropsychopharmacol. (2008) 18:826–34. 10.1016/j.euroneuro.2008.06.009 18674887 PMC2745315

[17] Howlett AC Barth F Bonner TI Cabral G Casellas P Devane WA. International union of pharmacology. XXVII. Classification of cannabinoid receptors. Pharmacol Rev. (2002) 54:161–202. 10.1124/pr.54.2.161 12037135

[18] Pacher P Bátkai S Kunos G The endocannabinoids system as an emerging target of pharmacotherapy. Pharamcol Rev. (2006) 58:389–462. 10.1124/pr.58.3.2 PMC2241751 16968947

[19] Mechoulam R Peters M Murillo-Rodriguez E Hanuš L. Cannabidiol – recent advances. Chem Biodivers. (2007) 4:1678–92. 10.1002/cbdv.200790147 17712814

[20] Mechoulam R Parker LA. The endocannabinoid system and the brain. Ann Rev Psychol. (2013) 64:21–47. 10.1146/annurev-psych-113011-143739 22804774

[21] Ashton C H. Pharmacology and effects of cannabis: a brief review. Br J Psychiatry. (2001) 178:101–6. 10.1192/bjp.178.2.101 11157422

[22] Fišar Z. Phytocannabinoids and endocannabinoids. Curr Drug Abuse Rev. (2009) 2:51–75. 10.2174/1874473710902010051 19630737

[23] Howlett AC. The cannabinoid receptors. Prostagl Other Lipid Mediat. (2002) 68:619–31. 10.1016/S0090-6980(02)00060-6 12432948

[24] Moreira FA Lutz B. The endocannabinoid system: emotion, learning and addiction. Addict Biol. (2008) 13:196–212. 10.1111/j.1369-1600.2008.00104.x 18422832

[25] Solinas M Goldberg SR Piomeli D The endocannabinoids system in brain reward processes. J Pharmacol. (2008) 154:369–83. 10.1038/bjp.2008.130 PMC2442437 18414385

[26] Covey DP Wenzel JM Cheer JF. Cannabinoid modulation of drug reward and the implications of marijuana legalization. Brain Res. (2014) 1628(Pt A):233–43. 10.1016/j.brainres.2014.11.034 25463025 PMC4442758

[27] Hillard CJ. Circulating endocannabinoids: from whence do they come and where are they going? Neuropsychopharmacology. (2017) 43:155–72. 10.1038/npp.2017.130 28653665 PMC5719092

[28] Witkin J Tzavara E Nomikos G. A role for cannabinoid CB1 receptors in mood and anxiety disorders. Behav Pharmacol. (2005) 16:315–31. 10.1097/00008877-200509000-00005 16148437

[29] McLaughlin RJ Hill MN Gorzalka BB. A critical role for prefrontocortical endocannabinoid signaling in the regulation of stress and emotional behavior. Neurosci Biobehav Rev. (2014) 42:116–31. 10.1016/j.neubiorev.2014.02.006 24582908

[30] Martin M Ledent C Parmentier M Maldonado R Valverde O. Involvement of CB1 cannabinoid receptors in emotional behaviour. Psychopharmacology. (2002) 159:379–87. 10.1007/s00213-001-0946-5 11823890

[31] Ashton CH Moore PB Gallagher P Young AH. Cannabinoids in bipolar affective disorder: a review and discussion of their therapeutic potential. J Psychopharmacol. (2005) 19:293–300. 10.1177/0269881105051541 15888515

[32] Hill M Gorzalka B. Pharmacological enhancement of cannabinoid CB1 receptor activity elicits an antidepressant-like response in the rat forced swim test. Eur Neuropsychopharmacol. (2005) 15:593–9. 10.1016/j.euroneuro.2005.03.003 15916883

[33] Adamczyk P Golda A McCreary A Filip M Przegalinski E. Activation of endocannabinoid transmission induces antidepressant-like effects in rats. J Physiol Pharmacol. (2008) 59:217–28. Available online at: http://www.jpp.krakow.pl/journal/archive/06_08/pdf/217_06_08_article.pdf 18622041

[34] Hillard C Weinlander K Stuhr K. Contributions of endocannabinoid signaling to psychiatric disorders in humans: genetic and biochemical evidence. Neuroscience. (2012) 204:207–29. 10.1016/j.neuroscience.2011.11.020 22123166 PMC3288440

[35] Marco EM Laviola G. The endocannabinoid system in the regulation of emotions throughout lifespan: a discussion on therapeutic perspectives. J Psychopharmacol. (2012) 26:150–63. 10.1177/0269881111408459 21693551

[36] Hell H Jager G Bossong M Brouwer A Jansma J Zuurman L. Involvement of the endocannabinoid system in reward processing in the human brain. Psychopharmacology. (2012) 219:981–90. 10.1007/s00213-011-2428-8 21822593 PMC3266503

[37] Hariri AR Gorka A Hyde LW Kimak M Halder I Ducci F Divergent effects of genetic variation in endocannabinoid signaling on human threat-and reward-related brain function. Biol Psychiatry. (2009) 66:9–16. 10.1016/j.biopsych.2008.10.047 19103437 PMC3215587

[38] Phan KL Angstadt M Golden J Onyewuenyi I Popovska A de Witt H. Cannabinoid modulation of amygdala reactivity to social signals of threat in humans. J Neurosci. (2008) 28:2313–9. 10.1523/JNEUROSCI.5603-07.2008 18322078 PMC2657360

[39] Pietrzak RH Huang Y Corsi-Travali S Zheng M Lin S Henry S. Cannabinoid type 1 receptor availability in the amygdala mediates threat processing in trauma survivors. Neuropsychopharmacology. (2014) 39:2519–28. 10.1038/npp.2014.110 24820537 PMC4207337

[40] Patterson DW Schmidt LA. Neuroanatomy of the human affective system. Brain Cognit. (2003) 52:24–6. 10.1016/S0278-2626(03)00005-8 12812801

[41] Pessoa L. A network model of the emotional brain. Trends Cogn. Sci. (2017) 21, 357–371. 10.1016/j.tics.2017.03.002 28363681 PMC5534266

[42] Etkin A Buchel C Gross JJ. The neural bases of emotion regulation. Nat Rev Neurosci. (2015) 16:693–700. 10.1038/nrn4044 26481098

[43] Frith CD Frith U. Social cognition in humans. Curr Biol. (2007) 17:724–32. 10.1016/j.cub.2007.05.068 17714666

[44] Lindquist KA Wager TD Kober H Bliss-Moreau E Barrett LF. The brain of emotion: a meta-analytic review. Behav Brain Sci. (2012) 35:121–42. 10.1017/S0140525X11000446 22617651 PMC4329228

[45] Glass M Dragunow M Faull RL. Cannabinoid receptors in the human brain: a detailed anatomical and quantitative autoradiographic study in the fetal, neonatal and adult human brain. Neuroscience. (1997) 77:299–318. 10.1016/S0306-4522(96)00428-9 9472392

[46] Mackie K Distribution of cannabinoid receptors in the central and peripheralnervous system. Handbook Exp Pharmacol. (2005) 168:299–325. 10.1007/3-540-26573-2_10 16596779

[47] Svizenska I Dubovy P Sulcova A Cannabinoid receptors 1 and 2 (CB1 and CB2), their distribution, ligands and functional involvement in nervous 80 system structure—a short review. Pharmacol Biochem Behav. (2008) 90:501–11. 10.1016/j.pbb.2008.05.010 18584858

[48] Terry GE Liow J Zoghbi SS Hirvonen J Farris AG Lerner A. Quantitation of cannabinoid CB1 receptors in healthy human brain using positron emission tomography and an inverse agonist radioligand. Neuroimage. (2009) 48:362–70. 10.1016/j.neuroimage.2009.06.059 19573609 PMC2730982

[49] Terry GE Hirvonen J Liow J-S Zoghbi SS Gladding R Tauscher JT. Imaging and quantitation of cannabinoid CB1 receptors in human and monkey brains using (18)F-labeled inverse agonist radioligands. J Nucl Med. (2010) 51:112–20. 10.2967/jnumed.109.067074 20008988 PMC2997525

[50] Heng L Beverley JA Steiner H Tseng KY. Differential developmental trajectories for CB1 cannabinoid receptor expression in limbic/associative and sensorimotor cortical areas. Synapse. (2011) 65:278–86. 10.1002/syn.20844 20687106 PMC2978763

[51] Hirvonen J Goodwin RS Li C Terry GE Zoghbi SS Morse C. Reversible and regionally selective downregulation of brain cannabinoid CB1 receptors in chronic daily cannabis smokers. Mol Psychiatry. (2012) 17:642–9. 10.1038/mp.2011.82 21747398 PMC3223558

[52] Ballard ME Bedi G de Wit H. Effects of delta-9-tetrahydrocannabinol on evaluation of emotional images. J Psychopharmacol. (2012) 26:1289–98. 10.1177/0269881112446530 22585232 PMC3664416

[53] Bossong MG van Hell HH Jager G Kahn RS Ramsey NF Jansma JM The endocannabinoid system and emotional processing: a pharmacological fMRI study with Δ9-tetrahydrocannabinol. Eur Neuropsychopharmacol. (2013) 23:1687–97. 10.1016/j.euroneuro.2013.06.009 23928295

[54] Hindocha C Freeman TP Schafer G Gardener C Das RK Morgan CJ Acute effects of delta-9-tetrahydrocannabinol, cannabidiol and their combination on facial emotion recognition: a randomized, double-blind, 66 placebo-controlled study in cannabis users. Eur Neuropsychopharmacol. (2015) 25:325–34. 10.1016/j.euroneuro.2014.11.014 25534187 PMC4398332

[55] Fusar-Poli P Crippa JA Bhattacharyya S Borgwardt SJ Allen P Martin-Santos R Distinct effects of {delta}9-tetrahydrocannabinol and 62 cannabidiol on neural activation during emotional processing. Arch Gen Psychiatry. (2009) 66:95–105. 10.1001/archgenpsychiatry.2008.519 19124693

[56] Fusar-Poli P Allen P Bhattacharyya S Crippa JA Mechelli A Borgwardt S. Modulation of effective connectivity during emotional processing by delta 9-tetrahydrocannabinol and cannabidiol. Int J Neuropsychopharmacol. (2010) 13:421–32. 10.1017/S1461145709990617 19775500

[57] Bhattacharyya S Falkenberg I Martin-Santos R Atakan Z Crippa JA Giampietro V. Cannabinoid modulation of functional connectivity within regions processing attentional salience. Neuropsychopharmacology. (2015) 40:1343–52. 10.1038/npp.2014.258 25249057 PMC4397391

[58] Bhattacharyya S Morrison PD Fusar-Poli P Martin-Santos R Borgwardt S Winton-Brown T. Opposite effects of delta-9- tetrahydrocannabinol and cannabidiol on human brain function and psychopathology. Neuropsychopharmacology. (2010) 35:764–74. 10.1038/npp.2009.184 19924114 PMC3055598

[59] Biswal BB Van Kylen J Hyde JS. Simultaneous assessment of flow and BOLD signals in resting-state functional connectivity maps. NMR Biomed. (1997) 10:165–70. 10.1002/(SICI)1099-1492(199706/08)10:4/5<165::AID-NBM454>3.0.CO;2-7 9430343

[60] Casey BJ Trainor RJ Orendi JL Schubert AB Nystrom LE Giedd JN. A developmental functional MRI study of prefrontal activation during performance of a go-no-go task. J Cogn Neurosci. (1997) 9:835–47. 10.1162/jocn.1997.9.6.835 23964603

[61] Joseph R. Environmental influences on neural plasticity, the limbic system, emotional development and attachment: a review. Child Psychiatry Hum Dev. (1999) 29:189–208. 10.1023/A:1022660923605 10080962

[62] Monk CS McClure EB Nelson EE Zarahn E Bilder RM Leibenluft E. Adolescent immaturity in attention-related brain engagement to emotional facial expressions. Neuroimage. (2003) 20:420–8. 10.1016/S1053-8119(03)00355-0 14527602

[63] Casey BJ Galvan A Hare TA. Changes in cerebral functional organization during cognitive development. Curr Opin Neurobiol. (2005) 15:239–44. 10.1016/j.conb.2005.03.012 15831409

[64] Casey B Tottenham N Liston C Durston S. Imaging the developing brain: what have we learned about cognitive development? Trends Cogn Sci. (2005) 9:104–10. 10.1016/j.tics.2005.01.011 15737818

[65] Liston C Watts R Tottenham N Davidson MC Niogi S Ulug AM. Frontostriatal microstructure modulates efficient recruitment of cognitive control. Cereb Cortex. (2006) 16:553–60. 10.1093/cercor/bhj003 16033925

[66] Choudhury S Blakemore S Charman T. Social cognitive development during adolescence. Soc Cogn Affect Neurosci. (2006) 1:165–74. 10.1093/scan/nsl024 18985103 PMC2555426

[67] Yurgelun-Todd D. Emotional and cognitive changes during adolescence. Curr Opin Neurobiol. (2007) 17:251–7. 10.1016/j.conb.2007.03.009 17383865

[68] Casey BJ Getz S Galvan A. The adolescent brain. Dev Rev. (2008) 28:62–77. 10.1016/j.dr.2007.08.003 18688292 PMC2500212

[69] Vink M Derks JM Hoogendam JM Hillegers M Kahn RS. Functional differences in emotion processing during adolescence and early adulthood. Neuroimage. (2014) 91:70–6. 10.1016/j.neuroimage.2014.01.035 24468408

[70] Blakemore SJ The social brain in adolescence. Nat Rev Neurosci. (2008) 9:267–77. 10.1038/nrn2353 18354399

[71] Braun K. The prefrontal-limbic system: development, neuroanatomy, function, and implications for socioemotional development. Clin Perinatol. (2011) 38:685–702. 10.1016/j.clp.2011.08.013 22107898

[72] Hariri AR Bookheimer SY Mazziotta JC. Modulating emotional responses: effects of a neocortical network on the limbic system. Neuroreport. (2000) 11:43–8. 10.1097/00001756-200001170-00009 10683827

[73] Durston S Davidson MC Tottenham N Galvan A Spicer J Fossella JA A shift from diffuse to focal cortical activity with development. Develop Neurosci. (2006) 1:1–8. 10.1111/j.1467-7687.2005.00454.x 16445387

[74] Kelly AMC Di Martino A Uddin LQ Shehzad Z Gee DG Reiss PT. Development of anterior cingulate functional connectivity from late childhood to early adulthood. Cereb Cortex. (2008) 19:640–57. 10.1093/cercor/bhn117 18653667

[75] Platt B Kamboj S Morgan CJ Curran HV. Processing dynamic facialaffect in frequent cannabis users: evidence of deficits in the speed of identifying emotional expressions. Drug Alcohol Depend. (2010) 112:27–32. 10.1016/j.drugalcdep.2010.05.004 21036306

[76] Huijbregts SC Griffith-Lendering MF Vollebergh WA Swaab H. Neurocognitive moderation of associations between cannabis use and psychoneuroticism. J Clin Exp Neuropsychol. (2014) 36:794–805. 10.1080/13803395.2014.943694 25116129

[77] Bayrakci A Sert E Zorlu N Erol A Saricicek A Mete L. Facial emotion recognition deficits in abstinent cannabis dependent patients. Compr Psychiatry. (2015) 58:160–4. 10.1016/j.comppsych.2014.11.008 25550274

[78] Hindocha C Wollenberg O Carter Leno V Alvarez BO Curran HV Freeman TP. Emotional processing deficits in chronic cannabis use: a replication and extension. J Psychopharmacol. (2014) 28:466–71. 10.1177/0269881114527359 24646810

[79] Gruber SA Rogowska J Yurgelun-Todd DA Altered affective responsein marijuana smokers: An FMRI study. Drug Alcohol Depend. (2009) 105:139–53. 10.1016/j.drugalcdep.2009.06.019 19656642 PMC2752701

[80] Cornelius JR Aizenstein HJ Hariri AR. Amygdala reactivity is inversely related to level of cannabis use in individuals with comorbid cannabis dependence and major depression. Addict Behav. (2010) 35:644–6. 10.1016/j.addbeh.2010.02.004 20189314 PMC2841401

[81] Spechler PA Orr CA Chaarani B Kan KJ Mackey S. Cannabis use in early adolescence: evidence of amygdala hypersensitivity to signals of threat. Develop Cogn Neurosci. (2015) 16:63–70. 10.1016/j.dcn.2015.08.007 26347227 PMC4801124

[82] Houck JM Bryan AD Ewing SF. Functional connectivity and cannabis use in high-risk adolescents. Am J Drug Alcohol Abuse. (2013) 39:414–23. 10.3109/00952990.2013.837914 24200211 PMC4070738

[83] Orr C Morioka R Behan B Datwani S Doucet M Ivanovic J. Altered resting-state connectivity in adolescent cannabis users. Am J Drug Alcohol Abuse. (2013) 39:372–81. 10.3109/00952990.2013.848213 24200207

[84] Behan B Connolly CG Datwani S Doucet M Ivanovic J Morioka R. Response inhibition and elevated parietal-cerebellar correlations in chronic adolescent cannabis users. Neuropharmacology. (2014) 84:131–7. 10.1016/j.neuropharm.2013.05.027 23791961

[85] Cheng H Skosnik PD Pruce BJ Brumbaugh MS Vollmer JM Fridberg DJ. Resting state functional magnetic resonance imaging reveals distinct brain activity in heavy cannabis users–a multi-voxel pattern analysis. J Psychopharmacol. (2014) 28:1030–40. 10.1177/0269881114550354 25237118 PMC4427512

[86] Pujol J Blanco-Hinoio L Batalla A López-Solà M Harrison BJ Soriano-Mas C. Functional connectivity alterations in brain networks relevant to self-awareness in chronic cannabis users. J Psychiatr Res. (2014) 51:68–78. 10.1016/j.jpsychires.2013.12.008 24411594

[87] Medina KL McQueeny T Nagel BJ Hanson KL Yang T Tapert SF. Prefrontal morphometry in abstinent adolescent marijuana users: Subtle gender effects. Addict Biol. (2009) 14:457–68. 10.1111/j.1369-1600.2009.00166.x 19650817 PMC2741544

[88] McQueeny TM Padula C Price J Medina KL Logan P Tapert SF. Gender effects on amygdala morphometry in adolescent marijuana users. Behav Brain Res. (2011) 224:128–34. 10.1016/j.bbr.2011.05.031 21664935 PMC3139567

[89] Schepis TS Desai RA Cavallo DA Smith AE Mcfetridge A Liss TB. Gender differences in adolescent marijuana use and associated psychosocial characteristics. J Addict Med. (2011) 5:65–73. 10.1097/ADM.0b013e3181d8dc62 21769049 PMC3359836

[90] Lisdahl KM Price JS. Increased marijuana use and gender predict poorer cognitive functioning in adolescents and emerging adults. J Int Neuropsychol Soc. (2012) 18:678–88. 10.1017/S1355617712000276 22613255 PMC3956124

[91] Shollenbarger SG Price J Wieser J Lisdahl KM. Impact of cannabis use on prefrontal and parietal cortex gyrification and surface area in adolescents and emerging adults. Develop Cogn Neurosci. (2015) 16:46–53. 10.1016/j.dcn.2015.07.004 26233614 PMC5289075

[92] Price JS McQueeny T Shollenbarger SG Browning EL Wieser J Lisdahl KM. Effects of marijuana use on prefrontal and parietal volumes and cognition in emerging adults. Psychopharmacology. (2015) 232:2939–50. 10.1007/s00213-015-3931-0 25921032 PMC4533900

[93] Lisdahl KM Tamm L Epstein JN Jernigan T Molina BSG Hinshaw SP. The impact of adhd persistence, recent cannabis use, and age of regular cannabis use onset on subcortical volume and cortical thickness in young adults. Drug Alcohol Depend. (2016) 161:135–46. 10.1016/j.drugalcdep.2016.01.032 26897585 PMC5289096

[94] Maple KE Thomas AM Kangiser MM Lisdahl KM. Anterior cingulate volume reductions in abstinent adolescent and young adult cannabis users: association with affective processing deficits. Psychiatry Res. (2019) 288:51–9. 10.1016/j.pscychresns.2019.04.011 31079000 PMC6548454

[95] Lopez-Larson MP Bogorodzki P Rogowska J McGlade E King JB Terry J. Altered prefrontal and insular cortical thickness in adolescent marijuana users. Behav Brain Res. (2011) 220:164–72. 10.1016/j.bbr.2011.02.001 21310189 PMC3073407

[96] Gilman JM Kuster JK Lee S Lee MJ Kim BW Makris N. Cannabis use is quantitatively associated with nucleus accumbens and amygdala abnormalities in young adult recreational users. J. Neurosci. (2014) 34:5529–39. 10.1523/JNEUROSCI.4745-13.2014 24741043 PMC3988409

[97] Schacht JP Hutchison KE Filbey FM. Associations between can- nabinoid receptor-1 (CNR1) variation and hippocampus and amyg- dala volumes in heavy *cannabis* users. Neuropsychopharmacology. (2012) 37:2368–76. 10.1038/npp.2012.92 22669173 PMC3442352

[98] Biswal BB Mennes M Zuo X-N Gohel S Kelly C Smith SM. Toward discovery science of human brain function. Proc Natl Acad Sci USA. (2010) 107:4734–9. 10.1073/pnas.0911855107 20176931 PMC2842060

[99] Di X Biswal BB. Dynamic brain functional connectivity modulated by resting-state networks. Brain Struct Funct. (2015) 220:37–46. 10.1007/s00429-013-0634-3 25713839 PMC3980132

[100] Fox MD Snyder AZ Zacks JM Raichle ME. The human brain is intrinsically organized into dynamic, anticorrelated functional networks. Proc Natl Acad Sci USA. (2005) 102:9673–8. 10.1073/pnas.0504136102 15976020 PMC1157105

[101] Fransson P. Spontaneous low-frequency BOLD signal fluctuations: an fMRI investigation of the resting-state default mode of brain function hypothesis. Hum Brain Mapp. (2005) 26:15–29. 10.1002/hbm.20113 15852468 PMC6871700

[102] Marcus DS Harms MP Snyder AZ Jenkinson M Wilson JA Glasser MF. Human Connectome Project informatics: quality control, database services, and data visualization. Neuroimage. (2013) 80:202–19. 10.1016/j.neuroimage.2013.05.077 23707591 PMC3845379

[103] Tomasi D Volkow ND. Functional connectivity density mapping. Proc Natl Acad Sci USA. (2010) 107:9885–90. 10.1073/pnas.1001414107 20457896 PMC2906909

[104] Yan C Liu D He Y Zou Q Zhu C Zuo X. Spontaneous brain activity in the default mode network is sensitive to different resting-state conditions with limited cognitive load. PLoS ONE. (2009) 4:5743. 10.1371/journal.pone.0005743 19492040 PMC2683943

[105] Patriat R Molloy EK Meier TB Kirk GR Nair VA Meyerand ME. The effect of resting condition on resting-state fMRI reliability and consistency: a comparison between resting with eyes open, closed and fixated. Neuroimage. (2013) 78:463–73. 10.1016/j.neuroimage.2013.04.013 23597935 PMC4003890

[106] Stonnington CM Tan G Kloppel S Chu C Draganski B Jack CRJr. Interpreting scan data acquired from multiple scanners: a study with Alzheimer's disease. Neuroimage. (2008) 39:1180–5. 10.1016/j.neuroimage.2007.09.066 18032068 PMC2225446

[107] Zuo XN Ehmke R Mennes M Imperati D Castellanos FX Sporns O. Network centrality in the human functional connectome. Cereb Cortex. (2012) 22:1862–75. 10.1093/cercor/bhr269 21968567

[108] Gogtay N Giedd JN Lusk L Hayashi KM Greenstein D Vaituzis AC. Dynamic mapping of human cortical development during childhood through earlyadult- hood. Proc Natl Acad Sci USA. (2004) 101:8174–9. 10.1073/pnas.0402680101 15148381 PMC419576

[109] American Psychiatric Association (2013). Diagnostic and Statistical Manual of Mental Disorders. 4th ed. Washington, DC: American Psychiatric Association.

[110] Sobell LC Maisto SA Sobell MB Cooper AM. Reliability of alcohol abusers' self-reports of drinking behavior. Behav Res Ther. (1979) 17:157–160. 10.1016/0005-7967(79)90025-1 426744

[111] Beck AT Steer RA Brown GK Manual for the Beck Depression Inventory-II. San Antonio, TX: Psychological Corporation (1996). 10.1037/t00742-000

[112] Storch EA Roberti JW Roth DA. Factor structure, concurrent validity, and internal consistency of the beck depression inventory-second edition in a sample of college students. Depress Anxiety. (2004) 19:187–9. 10.1002/da.20002 15129421

[113] Wechsler D Wechsler Abbreviated Scale of Intelligence. San Antonio, TX: The Psych Corp (1999).

[114] Wilkinson G Wide Range Achievement Test, (WRAT-4) Manual. 4th ed. Wilmington, DE: Wide Range, Inc (2006). 10.1037/t27160-000

[115] Willshire DK Kinsella G Prior M. Estimating WAIS-R IQ from the national adult reading test: a cross-validation. J Clin Exp Neuropsychol. (1991) 13:204–16. 10.1080/01688639108401038 1864911

[116] Manly JJ Jacobs DM Touradji P. Reading level attenuates differences in neuropsychological test performance between African American and White elders. J Int Neuropsychol Soc. (2002) 8:341–8. 10.1017/S1355617702813157 11939693

[117] Power JD Schlaggar BL Petersen SE. Recent progress and outstanding issues in motion correction in resting state fMRI. Neuroimage. (2015) 105:536–51. 10.1016/j.neuroimage.2014.10.044 25462692 PMC4262543

[118] Shirer WR Jiang H Price CM Ng B Greicius MD. Optimization of rs-fMRI Pre-processing for enhanced signal-noise separation, test-retest reliability, and group discrimination. Neuroimage. (2015) 117:67–79. 10.1016/j.neuroimage.2015.05.015 25987368

[119] Cox RW. AFNI: software for analysis and visualization of functional magnetic resonance neuroimages. Comp Biomed Res. (1996) 29:162–73. 10.1006/cbmr.1996.0014 8812068

[120] Smith SM Jenkinson M Woolrich MW Beckmann CF Behrens TEJ Johansen-Berg H. Advances in functional and structural MR image analysis and implementation as FSL. Neuroimage. (2004) 23:208–19. 10.1016/j.neuroimage.2004.07.051 15501092

[121] Power JD Barnes KA Snyder AZ Schlaggar BL Petersen SE. Spurious but systematic correlations in functional connectivity MRI networks arise from subject motion. Neuroimage. (2012) 59:2142–54. 10.1016/j.neuroimage.2011.10.018 22019881 PMC3254728

[122] Dale AM Fischl B Sereno MI. Cortical surface-based analysis. I. Segmentation and surface reconstruction. Neuroimage. (1999) 9:179–94. 10.1006/nimg.1998.0395 9931268

[123] Klein A Tourville J. 101 labeled brain images and a consistent human cortical labeling protocol. Front Neursci. (2012) 6:171. 10.3389/fnins.2012.00171 23227001 PMC3514540

[124] Fischl B Salat DH Busa E Albert M Dieterich M Haselgrove C. Whole brain segmentation: automated labeling of neuroanatomical structures in the human brain. Neuron. (2002) 33:341–55. 10.,1016/S0896-6273(02)00569-X 11832223

[125] Benjamini Y Hochberg Y Controlling the false discovery rate: a practical and powerful approach to multiple testing. J R Stat Soc. (1995) 57:289–300. 10.1111/j.2517-6161.1995.tb02031.x

[126] Xia M Wang J He Y. BraiNet viewer: a network visualization tool for human brain connectomics. PLoS ONE. (2013) 8:e68910. 10.1371/journal.pone.0068910 23861951 PMC3701683

[127] Eldreth DA Matochik JA Cadet JL Bolla KI. Abnormal brain activity in prefrontal brain regions in abstinent marijuana users. Neuroimage. (2004) 23:914–20. 10.1016/j.neuroimage.2004.07.032 15528091

[128] Schweinsburg AD Schweinsburg BC Cheung EH Brown GG Brown SA Tapert SF fMRI response to spatial working memory in adolescents with comorbid marijuana and alcohol use disorder. Drug Alcohol Depend. (2005) 79:201–10. 10.1016/j.drugalcdep.2005.01.009 16002029 PMC2270678

[129] Cheng L Yakupov R Cloak C Ernst T Marijuana use is associated with a reorganized visual-attention network and cerebellar hypoactivation. Brain. (2006) 129:1096–112. 10.1093/brain/awl064 16585053

[130] Tapert S Schweinsburg A Drummond S Paulus M Brown S Yang T. Functional MRI of inhibitory processing in abstinent adolescent marijuana users. Psychopharmacology. (2007) 194:173–83. 10.1007/s00213-007-0823-y 17558500 PMC2269705

[131] Hester R Nestor L Garavan H. Impaired error awareness and anterior cingulate cortex hypoactivity in chronic cannabis users. Neuropsychopharmacology. (2009) 34:2450–8. 10.1038/npp.2009.67 19553917 PMC2743772

[132] Schweinsburg AD Schweinsburg BC Lisdahl Medina K McQueeny T Brown SA Tapert SF. The influenct of recency of use on fMRI reponse during spatial working memory in adolesecent marijuana users. J Psychoactive Drugs. (2010) 42:401–12. 10.1080/02791072.2010.10400703 21053763 PMC3016644

[133] Wesley MJ Hanlon CA Porrino LJ. Poor decition-making by chronic marijuana users is associated with decreased functional responsiveness to negative consequences. Psychiatry Res. (2011) 191:51–9. 10.1016/j.pscychresns.2010.10.002 21145211 PMC3125637

[134] Sneider JT Gruber SA Rogowska J Silveri MM Yurgelun-Todd DA. A preliminary study of functional brain activation among marijuana users during performance of a virtual water maze task. J. Addict. (2013) 2013:461029. 10.1155/2013/461029 23951549 PMC3742334

[135] Nieuwenhuis S Ridderinkhof KR Blom J Band GP Kok A. Error-related brain potentials are differentially related to awareness of response errors: evidence from an antisaccade task. Psychophysiology. (2001) 38:752–60. 10.1111/1469-8986.3850752 11577898

[136] Klein TA Endrass T Kathmann N Neumann J von Cramon DY Ullsperger M. Neural correlates of error awareness. Neuroimage. (2007) 34:1774–81. 10.1016/j.neuroimage.2006.11.014 17185003

[137] Hester R Foxe JJ Molholm S Shpaner M Garavan H. Neural mechanisms involved in error processing: a comparison of errors made with and without awareness. Neuroimage. (2005) 27:602–8. 10.1016/j.neuroimage.2005.04.035 16024258

[138] O'Connell RG Dockree PM Bellgrove MA Kelly SP Hester R Garavan H. The role of cingulate cortex in the detection of errors with and without awareness: a high-density electrical mapping study. Eur J Neurosci. (2007) 25:2571–9. 10.1111/j.1460-9568.2007.05477.x 17445253

[139] Beckman M Johansen-Berg H Rushworth MFS Connectivity-based parcellation of human cingulate cortex and its relation to functional specialization. J Neurosci. (2009) 29:1175–90. 10.1523/JNEUROSCI.3328-08.2009 19176826 PMC6665147

[140] Devinsky O Morrell MJ Vogt BA. Contributions of anterior cingulate cortex to behaviour. Brain. (1995) 118(Pt 1): 279–306. 10.1093/brain/118.1.279 7895011

[141] Bush G Luu P Posner MI. Cognitive and emotional influences in anterior cingulate cortex. Trends Cogn Sci. (2000) 4:215–22. 10.1016/S1364-6613(00)01483-2 10827444

[142] Seeley WW Menon V Schatzberg AF Keller J Glover GH Kenna H. Dissociable intrinsic connectivity networks for salience processing and executive control. J Neurosci. (2007) 27:2349–56. 10.1523/JNEUROSCI.5587-06.2007 17329432 PMC2680293

[143] Cooney RE Joormann J Atlas LY Eugène F Gotlib IH. Remembering the good times: neural correlates of affect regulation. Neuroreport. (2007) 18:1771–4. 10.1097/WNR.0b013e3282f16db4 18090309

[144] Etkin A Egner T Kalisch R. Emotional processing in anterior cingulate and medial prefrontal cortex. Trends Cogn Sci. (2011) 15:85–93. 10.1016/j.tics.2010.11.004 21167765 PMC3035157

[145] Di Pellegrino G Ciaramelli E Làdavas E. The regulation of cognitive control following rostral anterior cingulate cortex lesion in humans. J Cogn Neurosci. (2007) 19:275–86. 10.1162/jocn.2007.19.2.275 17280516

[146] Troup LJ Bastidas S Nguyen MT Andrzejewski JA Bowers M Nomi JS. An event-related potential study on the effects of cannabis on emotion processing. PLoS ONE. (2016) 11:e0149764. 26926868 10.1371/journal.pone.0149764 PMC4772908

[147] Botteron KN Raichle ME Drevets WC Heath AC Todd RD. Volumetric reduction in left subgenual prefrontal cortex in early onset depression. Biol Psychiatry. (2002) 51:342–4. 10.1016/S0006-3223(01)01280-X 11958786

[148] Hajek T Kozeny J Kopecek M Alda M Höschl C. Reduced subgenual cingulate volumes in mood disorders: a meta-analysis. J Psychiatry Neurosci. (2008) 33:91–9. 18330455 PMC2265308

[149] Greicius MD Flores BH Menon V Glover GH Solvason HB Kenna H. Resting-state functional connectivity in major depression: abnormally increased contributions from subgenual cingulate cortex and thalamus. Biol Psychiatry. (2007) 62:429–37. 10.1016/j.biopsych.2006.09.020 17210143 PMC2001244

[150] Mayberg H Lozano AM Voon V McNeely HE Seminowicz D Hamani C. Deep brain stimulation for treatment-resistant depression. Neuron. (2005) 45:651–60. 10.1016/j.neuron.2005.02.014 15748841

[151] Drevets WC Savitz J Trimble M. The subgenual anterior cingulate cortex in mood disorders. CNS Spectrums. (2009) 13:663–81. 10.1017/S1092852900013754 18704022 PMC2729429

[152] Strikwerda-Brown C Davey CG Whittle S Allen NB Byrne ML Schwartz OS. Mapping the relationship between subgenual cingulate cortex functional connectivity and depressive symptoms across adolescence. Soc Cogn Affect Neurosci. (2015) 10:961–8. 10.1093/scan/nsu143 25416726 PMC4483565

[153] Etkin A Wager TD. Functional neuroimaging of anxiety: a meta-analysis of emotional processing in PTSD, social anxiety disorder, and specific phobia. Am J Psychiatry. (2007) 164:1476–88. 10.1176/appi.ajp.2007.07030504 17898336 PMC3318959

[154] Pizzagalli D Pascual-Marqui RD Nitschke JB Oakes TR Larson CL Abercrombie HC. Anterior cingulate activity as a predictor of degree of treatment response in major depression: evidence from brain electrical tomography analysis. Am J Psychiatry. (2001) 158:405–15. 10.1176/appi.ajp.158.3.405 11229981

[155] Moore TH Zammit S Lingford-Hughes A Barnes TR Jones PB Burke M. Cannabis use and risk of psychotic or affective mental health outcomes: a systematic review. Lancet. (2007) 370:319–28. 10.1016/S0140-6736(07)61162-3 17662880

[156] Troup LJ Andrzejewski JA Braunwalder JT Torrence RD. The relationship between cannabis use and measures of anxiety and depression in a sample of college campus cannabis users and non-users post state legalization in Colorado. PeerJ. (2016) 4:e2782. 10.7717/peerj.2782 27957402 PMC5149055

[157] Shollenbarger SG Price J Wieser J Lisdahl KM. Poorer frontolimbic white matter integrity is associated with chronic cannabis use, *FAAH* genotype, and increased depressive and apathy symptoms in adolescents and young adults. Neuroimage Clin. (2015) 8:117–25. 10.1016/j.nicl.2015.03.024 26106535 PMC4473294

[158] Prescot AP Locatelli AE Renshaw PF Yurgelun-Todd DA. Neurochemical alterations in adolescent chronic marijuana smokers: a proton MRS study. Neuroimage.(2011) 57:69–75 10.1016/j.neuroimage.2011.02.044 21349338 PMC3101285

[159] Prescot AP Renshaw PF Yurgelun-Todd DA. γ-amino butyric acid and glutamate abnormalities in adolescent chronic marijuana smokers. Drug Alcohol Depend. (2013) 129:232–9. 10.1016/j.drugalcdep.2013.02.028 23522493 PMC4651432

[160] Duncan NW Enzi B Wiebking C Northoff G. Involvement of glutamate in rest-stimulus interaction between perigenual and supragenual anterior cingulate cortex: a combined fMRI-MRS study. Hum Brain Mapp. (2011) 32:2172–82. 10.1002/hbm.21179 21305662 PMC6870441

[161] Feldstein Ewing SW McEachern AD Yezhuvath U Bryan AD Hutchison KE Filbey FM. Integrating brain and behavior: evaluating adolescetns' response to a cannabis intervention. Psychol Addict Behav. (2013) 27:510–25. 10.1037/a0029767 22925010 PMC6693491

[162] Paulus MP Stewart JL Haase L. Treatment approaches for interoceptive dysfunctions in drug addiction. Front Psychiatry. (2013) 4:137. 10.3389/fpsyt.2013.00137 24151471 PMC3798869

[163] Boys A Marsden J Strang J. Understanding reasons for drug use amongst young people: a functional perspective. Health Educ Res. (2001) 16:457–69. 10.1093/her/16.4.457 11525392

[164] McCarty CA Rhew IC Murowchick E McCauley E Stoep AV. Emotional health predictors of substance use initiation during middle school. Psychol Add Behav. (2012) 26:351–7. 10.1037/a0025630 21988479 PMC3262933

[165] Lopez B Wang W Schwartz SJ Prado G Huang S Brown CH. School, family, and peer factors and their association with substance use in hispanic adolescents. J Prim Prevent. (2009) 30:622–41. 10.1007/s10935-009-0197-5 19949868 PMC2916637

[166] Prado G Huang S Schwartz SJ Maldonado-Molina MM Bandiera FC de la Rosa M. What accounts for differences in substance use among U.S.-born and immigrant hispanic adolescents?: results from a longitudinal prospective cohort study. J Adolesc Health. (2009) 45:118–25. 10.1016/j.jadohealth.2008.12.011 19628137 PMC3466101

[167] Connell CM Gilreath TD Aklin WM Brex RA. Social-ecological influences on patterns of substance use among non-metropolitan high school students. Am J Commun Psychol. (2010) 45:36–48. 10.1007/s10464-009-9289-x 20077132 PMC3970316

[168] Kiesner J Poulin F Dishion TJ. Adolescent substance use with friends: moderating and mediating effects of parental monitoring and peer activity contexts. Merrill Palmer Q. (2010) 56:529–56. 10.1353/mpq.2010.0002 21165170 PMC3002110

[169] Branstetter SA Low S Furman W. The influence of parents and friends on adolescent substance use: a multidimensional approach. J Substance Use. (2011) 16:150–60. 10.3109/14659891.2010.519421 21747736 PMC3132133

[170] Karriker-Jaffe KJ. Areas of disadvantage: a systematic review of effects of area-level socioeconomic status on substance use outcomes. Drug Alcohol Rev. (2011) 30:84–95. 10.1111/j.1465-3362.2010.00191.x 21219502 PMC3057656

[171] Van Ryzin MJ Fosco GM Dishion TJ. Family and peer predictors of substance use from early adolescence to early adulthood: an 11-year prospective analysis. Addict Behav. (2012) 37:1314–24. 10.1016/j.addbeh.2012.06.020 22958864 PMC3459354

[172] Eisenberg ME Toumbourou JW Catalano RF Hemphill SA. Social norms in the development of adolescent substance use: a longitudinal analysis of the international youth development study. J Youth Adolesc. (2014) 43:1486–97. 10.1007/s10964-014-0111-1 24633850 PMC4130778

[173] Sitnick S Shaw DS Hyde L. Precursors of adolescent substance use from early childhood and early adolescence: testing a developmental cascade model. Develop Psychopathol. (2014) 26:125–40. 10.1017/S0954579413000539 24029248 PMC3864122

[174] Unger JB. Cultural influences on substance use among hispanic adolescents and young adults: findings from project RED. Child Develop Perspect. (2014) 8:48–53. 10.1111/cdep.12060 24729791 PMC3979561

[175] Bacio GA Estrada Y Huang S Martínez M Sardinas K Prado G. Ecodevelopmental predictors of early initiation of alcohol, tobacco, and drug use among hispanic adolescents. J Sch Psychol. (2015) 53:195–208. 10.1016/j.jsp.2015.02.001 26054814 PMC4461835

[176] Burgdorf JR Kilmer B Pacula RL. Heterogeneity in the composition of marijuana seized in California. Drug Alcohol Depend. (2011) 117:59–61. 10.1016/j.drugalcdep.2010.11.031 21288662 PMC3118261

[177] ElSohly MA Ross SA Mehmedic Z Arafat R Yi B Banahan BFIII. Potency trends of delta9-THC and other cannabinoids in confiscated marijuana from 1980-1997. J Forensic Sci. (2000) 45:24–30. 10.1520/JFS14636J 10641915

[178] Mehmedic Z Chandra S Slade D Denham H Foster S Patel AS. Potency trends of Δ9-THC and other cannabinoids in confiscated cannabis preparations from 1993 to 2008. J Forensic Sci. (2010) 55:1209–17. 10.1111/j.1556-4029.2010.01441.x 20487147

[179] Gage S Hickman M Heron J Munafò MR Lewis G Macleod J. Associations of cannabis and cigarette use with depression and anxiety at age 18: findings from the Avon Longitudinal study of parents and children. PLoS ONE. (2015) 10:e0122896. 10.1371/journal.pone.0122896 25875443 PMC4395304

[180] Kedzior KK Laeber LT. A positive association between anxiety disorders and cannabis use or cannabis use disorders in the general population- a meta-analysis of 31 studies. BMC Psychiatry. (2014) 14:1–39. 10.1186/1471-244X-14-136 24884989 PMC4032500

[181] Chadwick B Miller ML Hurd YL. Cannabis use during adolescent development: suspectibility to psychiatric illness. Front Psychiatry. (2013) 4:129. 10.3389/fpsyt.2013.00129 24133461 PMC3796318

[182] Lisdahl KM Gilbart ER Wright NE Shollenbarger SG. Dare to delay? The impacts of adolescent alcohol and marijuana use onset on cognition, brain structure, and function. Front Psychiatry. (2013) 4:53. 10.3389/fpsyt.2013.00053 23847550 PMC3696957

[183] Lisdahl KM Wright NE Medina-Kirchner C Maple KE Shollenbarger S. Considering cannabis: the effects of regular cannabis use on neurocognition in adolescents and young adults. Curr Addict Rep. (2014) 1:144–56. 10.1007/s40429-014-0019-6 25013751 PMC4084860

[184] Wade T Wallace AL Swartz AM Lisdahl KM Aerobic fitness level moderates the association between cannabis use and executive functioning and psychomotor speed following abstinence in adolescents and young adult. J Int Neuropsychol Soc. (2018) 25:134–45. 10.1017/S1355617718000966 30474579 PMC6374167

